# Closed-Loop Control of Chemical Injection Rate for a Direct Nozzle Injection System

**DOI:** 10.3390/s16010127

**Published:** 2016-01-20

**Authors:** Xiang Cai, Martin Walgenbach, Malte Doerpmond, Peter Schulze Lammers, Yurui Sun

**Affiliations:** 1 School of Information Science and Technology, Beijing Forestry University, Beijing 100083, China; 2 Department of Agricultural Engineering, University of Bonn, 53115 Bonn, Germany; martin_walgenbach@t-online.de (M.W.); malte@uni-bonn.de (M.D.); lammers@uni-bonn.de (P.S.L.); 3 College of Information & Electrical Engineering, China Agricultural University, Beijing 100083, China; pal@cau.edu.cn

**Keywords:** variable-rate application, closed-loop control, pulse width modulation, direct nozzle injection, thermodynamic flowmeter

## Abstract

To realize site-specific and variable-rate application of agricultural pesticides, accurately metering and controlling the chemical injection rate is necessary. This study presents a prototype of a direct nozzle injection system (DNIS) by which chemical concentration transport lag was greatly reduced. In this system, a rapid-reacting solenoid valve (RRV) was utilized for injecting chemicals, driven by a pulse-width modulation (PWM) signal at 100 Hz, so with varying pulse width the chemical injection rate could be adjusted. Meanwhile, a closed-loop control strategy, proportional-integral-derivative (PID) method, was applied for metering and stabilizing the chemical injection rate. In order to measure chemical flow rates and input them into the controller as a feedback in real-time, a thermodynamic flowmeter that was independent of chemical viscosity was used. Laboratory tests were conducted to assess the performance of DNIS and PID control strategy. Due to the nonlinear input–output characteristics of the RRV, a two-phase PID control process obtained better effects as compared with single PID control strategy. Test results also indicated that the set-point chemical flow rate could be achieved within less than 4 s, and the output stability was improved compared to the case without control strategy.

## 1. Introduction

Previous studies have pointed out that there are advantages to realizing site-specific and variable-rate application of pesticides [1,2,3]. Broadcast application of chemicals without concentration variance and spatial selection results in fields being sprayed without distinction. On the one hand, over-application of chemicals not only increases the cost of crop production, but also increases the risk of environmental contamination and the exposure hazards to operators. On the other hand, insufficient application of chemical may cause a decrease of crop yield, or make the weeds chemical-resistant.

Conventional boom sprayers mix a measured quantity of pesticide with a defined amount of carrier in a tank. Chemical application rates can only be varied by changing the liquid delivery pressure, but the pressure changes also unexpectedly affect the droplet size spectra and spray distribution pattern of the nozzles [4]. However, by using a direct injection system, chemical and carrier are kept separately and mixed together when sprayed. The delivery pressure of carrier stream is maintained at the same level, as are the pressure at the nozzles, the droplet size spectrum and the spray distribution pattern [5]. Through changing the chemical injection volume, the chemical application rate can be changed.

GopalaPillai *et al.* tested an electrical flow control system for site-specific herbicide applications and its steady state performance [6]. Solenoid valves were used and controlled by a PWM signal in the system. The chemical flow rate was varied by changing the duty cycle of the PWM signal from 10% to 100%. However, this was an open-loop system. The precision and stabilization of chemical flow rate were not discussed. Han *et al.* also tested a DIS with solenoids controlled by a PWM signal in an open-loop control system [7]. They found the flow rate control error ranged from −15% to +12%.

Effective flow rate control requires fast and accurate metering of chemicals for the direct injection sprayer, which could be accomplished by using a positive driven pump or a closed–loop control system with the help of flowmeters [8]. Frost *et al.* developed a metering system in which a metered flow of water was used to control the flow rate of chemical [9]. That was a kind of central direct injection system (CDIS), where chemical was injected into the system downstream from the main carrier tank and prior to branching of the distribution hoses carrying the solution to different boom sections. The advantage of that system is that the chemical was metered by a closed-loop contoller so that it could provide a wide range of chemical dose rates precisely, while the disadvantage was the long lag time from a change in the chemical flow rate to the corresponding changes in its concentration at the spraying points [10].

To solve the long lag time problem, Vondricka and Schulze Lammers proposed a direct nozzle injection system (DNIS) concept, in which the point where the chemical was injected into the system was changed to be at the nozzles [11]. This improvement shortened the chemical flow path and hence reduced the lag time significantly. Subsequently, their team focused on many aspects of this DNIS. Vondricka addressed on the problem of mixture homogeneity and Doerpmund assessed the cleanability of this DNIS [12,13,14]. However, there is still a need to investigate the performance for DNIS metering and chemical flow rate control, which is the premise for realizing site-specific and variable-rate application of chemicals.

The objective of this article was to test the injection uniformity of RRVs within a boom section of the DNIS, to present a closed-loop control method to meter and stabilize the chemical application rate with the help of a thermodynamic flowmeter, and to evaluate the performance of the control system and the DNIS by an EC sensor.

## 2. Materials and Methods

### 2.1. Description of RRV

**Figure 1:**
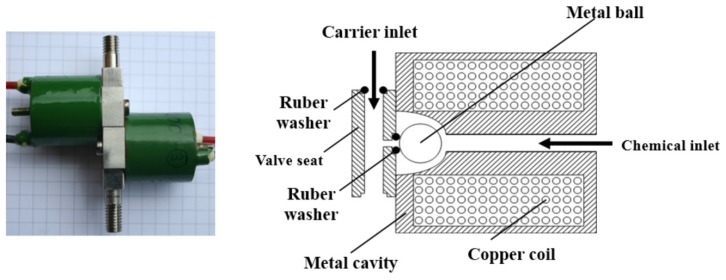
Structure of the RRV.

A new rapid reacting magnetic valve design was developed to inject pesticides. A schematic of its structure is shown in Figure 1. It mainly consists of a cylindrical metal cavity, an induction copper coil (resistance: *ca.* 3~5 Ω), metal ball, valve seat and rubber washers.

When electric current flows through the copper coil, a strong magnetic field is generated inside the metal cavity based on the electromagnetic induction principle, then the metal ball is pushed aside by the magnetic field. Thus an access for the chemical from the inlet to the orifice of the valve seat is formed, through which pressurized chemical can be injected. If there is no electric current in the coil, the pressurized chemical pushes the metal ball onto the valve seat causing a tight contact between the ball and the rubber washer, which finally shut down the flux of chemical. In this case, RRV is switched off.

The RRV is powered by a PWM signal at a frequency of 100 HZ generated by a FPGA controller. The pulse width during which the RRV remains open in one PWM period determines the injection time and hence the injection volume of chemical. Thus, by adjusting the length of the pulse width, the chemical injection rate could be varied.

### 2.2. Experiment Setup

**Figure 2:**
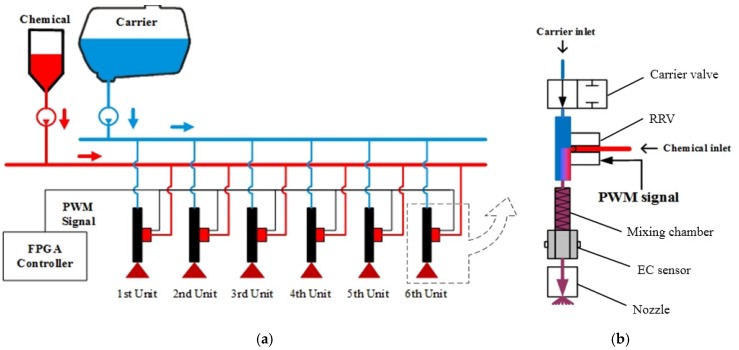
(**a**) Schematic structure of the experimental setup with a boom section; (**b**) one of the injection units.

The proposed DNIS was 21 m of length, and was equipped with 42 direct injection units which were divided into seven boom sections. Within one section, six direct injection units were controlled by a same PWM signal as shown in Figure 2a. Figure 2b shows the structure of one direct injection unit, which includes a carrier valve, a RRV, a mixing chamber, an EC sensor, and a spray nozzle.

**Figure 3:**
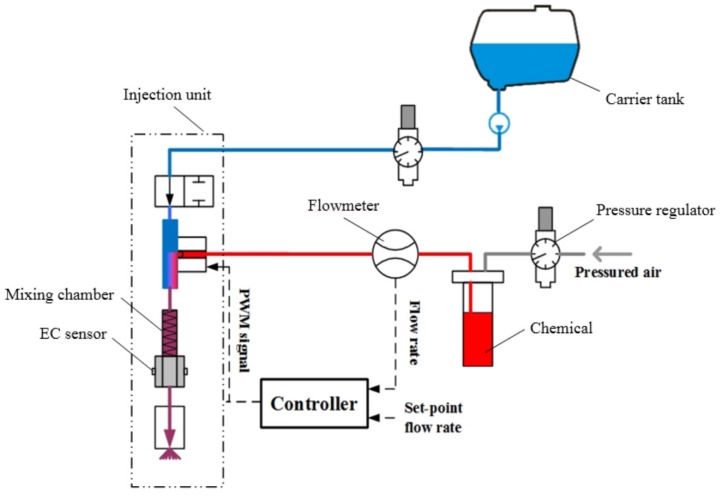
Setup for controlling pesticide injection rate by closed-loop method.

Figure 3 shows test bench to implement our closed-loop control strategy for metering the chemical injection rate of the RRV. It is composed of a direct injection unit, flowmeter, two pressure regulators, and a controller. The EC sensor mounted between mixing chamber and nozzle in injection unit was used to indicate the concentration of the spraying solution, it could also be used to verify the control effect.

The flowmeter is based on the thermodynamic principle. When a liquid medium flows through, the sensor generates a heat pulse internally. The heat is then conducted away by the medium flux resulting in a cooling down of the sensor. The temperature within the sensor is measured and compared with the temperature of the medium, and the flow rate can be derived from the temperature difference. Theoretically, the flowmeter is independent of the viscosity of the agricultural chemicals.

The chemical applied in the experiments was not real, and it was simulated by a solution consisting of (in proportion by weight) 10% LUVITEC^®^ powder, 3% salt and 87% water. The LUVITEC^®^ powder was used as additive to adjust the solution viscosity in the range of 230–240 mPa·s. Salt made the solution electrical conductive, so the EC sensor could detect the concentration of the solution. The more electroconductive the solution at the nozzle was, the more chemical the RRV injected. Moreover, the stability of EC values indicated the stability of the chemical flow rate.

### 2.3. Uniformity Test of 6 RRVs in One Boom Section

Within a boom section, six injection units are controlled by one PWM signal (see Figure 2a), so they are expected to inject chemical in a same rate, but because the chemical loses pressure along the flux direction in the boom, this results in the pressures at injection units being different, and there exist individual differences among these RRVs, so the chemical injection rates are not consistent in practice. Therefore experiments to assess the uniformity of six RRVs in one boom section when they work in an open control loop were conducted. In this experiment, water and chemical pressure were adjusted at 3 bar and 7 bar, respectively, and the pulse width of the PWM signal was set at 1000 μs. The volumes of chemical injected by the six injection units in 3 min were sampled three times repeatedly, and the the average injected volume per minute and the standard deviation were calculated as the injection rate and error bar, respectively.

### 2.4. Calculating the Range of Chemical Injection Rate

Assume that the DNIS (with a boom of 21 m in length and with 42 injection units) was used for spraying in a field, and each injection unit was supposed to inject chemical in a same rate of *r* mL/min. If the tractor drove with speed of *v* m/h, after *t* hours, the sprayed area would be *S* ha and the total volume of chemical sprayed would be *W* mL. The the following equations could be derived:

(1) 42 × *rt* × 60 = *W*

(2) 21 × *vt* = 10000 × *S*

Equation (1) divided by Equation (2) gives the chemical injection rate r:

(3) $$r=\frac{vW}{1200000S}$$

Empirically, the tractor speed *v* ranged from 6 km/h to 10 km/h, chemical per hectare needed in the field (*W/S*) was assumed from 20 mL/ha to 4000 mL/ha. Accoording to Equation (3), the injection rate of each RRV should range from 0.1 to 33.3 mL/min, which could cover most spraying applications.

### 2.5. Closed-Loop Control

The chemical injection rate from the RRV is susceptible to factors such as the delivery pressure of both carrier and chemical, chemical viscosity, and any unexpected external disturbances, so the actual flow rate injected by the RRV deviates from the desired value while spraying because it is not possible to keep the operation conditions constant. The deviation would not be corrected automatically in a system with an open-loop control strategy, but a closed-loop controller could compensate for the deviation and keep the flow rate stable, thus realizing a more precise application of chemical.

**Figure 4:**
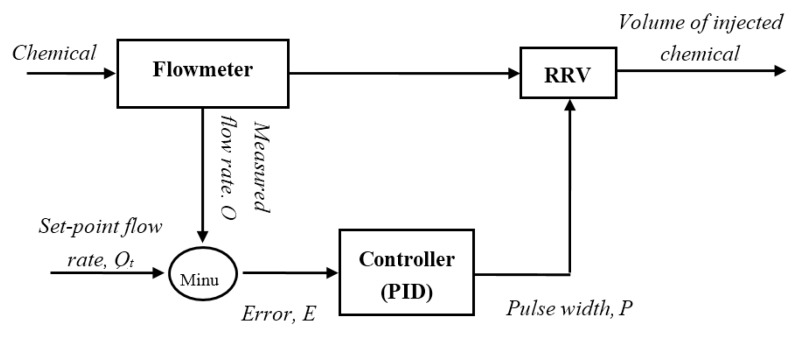
Block diagram of the closed-loop control.

Figure 4 shows the block diagram of a closed-loop control system for the chemical injection rate, based on the PID method. Symbol *E* represents the deviation between the set-point flow rate *Q_t_* and the actual flow rate *Q* measured by a flowmeter. The controller adjusts the pulse width *P* of the PWM signal to minimize *E*.

### 2.6. Experimental Procedures

The following experiments were performed:

1. The RRV was tested, and the relationship between the input pulse width *P* and the output chemical injection rate was investigated; in this experiment, the water pressure was set at 3 bar, and the air pressure for pushing the chemical was adjusted to 6, 7 and 8 bar to obtain the input-output characteristics of the RRV under three pressure differences and hence to compare the optimal operating conditions for the RRV.
2. The uniformity of six RRVs controlled by the same PWM signal (pulse width was 1000 μs) within one boom section was tested.
3. The flowmeter was calibrated. The amount of injected chemical in 3 min was weighed three times, an the average value per minute was calculated as the injection rate.
4. A single PID module was applied in the control system at first. Subsequently a two-phase PID control strategy was used that managed to improve the control effect in accordance with the input-output characteristic of the RRV.

## 3. Results and Discussion

### 3.1. Tests of the RRV

**Figure 5:**
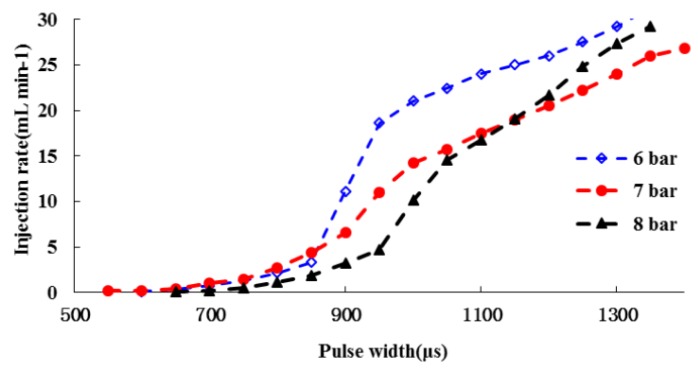
Injection rate of RRV under three air pressures, calibrated by using 10% LUVITEC^®^ solution.

Figure 5 shows the injection rate of the RRV as a function of the pulse width under three pressure differences. Through experimental tests, it was determined that an air pressure of 7 bar for pushing the chemical and a water pressure of 3 bar were the optimal choice for the best performance of the RRV and nozzle. The curves demonstrate that RRV has a large injection rate range from less than 0.1 mL/min to more than 30 mL/min, that meets the requirements as calculated previously. The curve under a pressure difference of 4 bar is smoother than those of 3 bar and 5 bar, which means the input-output characteristics of the RRV in this case is more linear than the other cases, and the normally linear system is easier to control.

### 3.2. Uniformity Test of Six RRVs within One Boom Section

**Figure 6:**
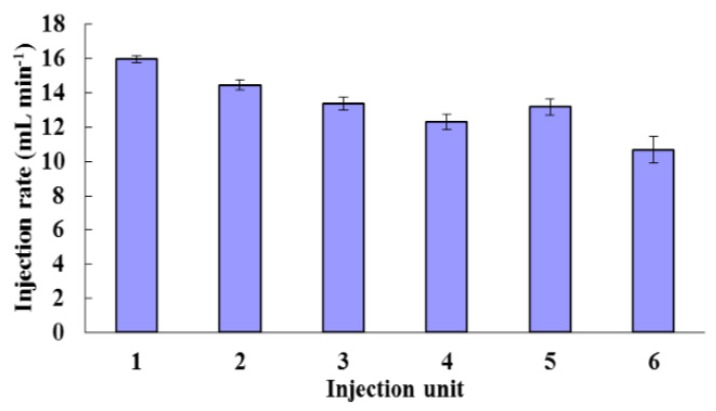
Injection rates of 6 RRVs within one boom section, when PWM signal was with pulse width 1000 μs.

Figure 6 shows that, under the given experimental conditions: (1) the injection rates of six RRVs within one boom section are roughly equal, and around 14 mL/min; (2) however, along the direction of chemical flow in the boom the injection rate of each RRV has a trend to slightly decrease, which is probably due to the loss of chemical pressure at the inlet of the RRVs, which leads to the RRVs not being operated at the recommended pressure; (3) the error bars representing the standard deviations, (which range from 0.2 to 0.77 mL/min), emphasise the unstable flows injected by the RRVs.

The error bars increase from injection unit 1 to 6 illustrates that the further away the injection unit is from the chemical inlet of the boom, the more unstable the flow rate is. Based on this, it could be concluded that, with an open control strategy, it is hard for the DNIS to meter and inject chemical precisely, even under stable conditions, but metering and injecting the chemical precisely is the premise for site-specific and variable-rate application of chemicals, so closed-loop control is necessary for this DNIS.

### 3.3. Calibration of the Thermodynamic Flowmeter

**Figure 7:**
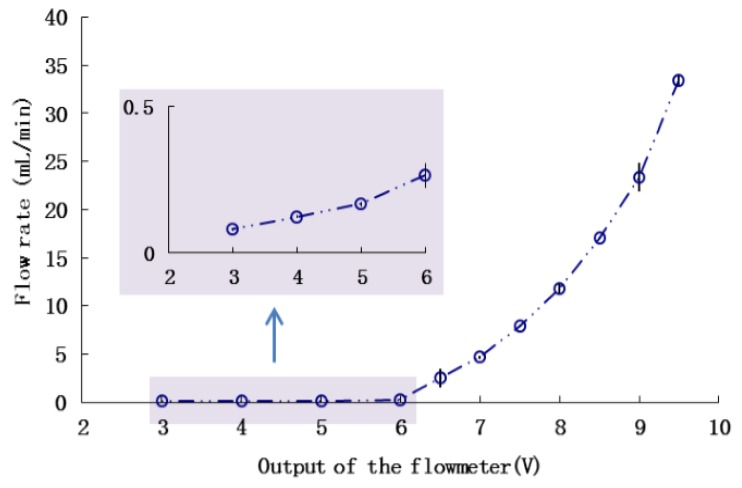
Calibration of the flowmeter.

The thermodynamic flowmeter has a wide measurement range, which is determined by adjusting the coefficient of amplification manually. In accordance with range of RRV injection rates, the measurement scale was specified and the calibration curve was obtained as shown in Figure 7. Although this thermodynamic flowmeter was independent of chemical viscosity, it was affected by the thermal conductivity of the liquid medium. Thus, before a different type of chemical can be applied, a specific calibration is required in advance.

### 3.4. Tuning the PID Parameters

According to the Ziegler-Nichols method introduced by Frost [9], the transfer function of RRV could be simplified as below:

(4) $$\frac{Q(s)}{P(s)}=\frac{{ke}^{-{sT}_{1}}}{1+{sT}_{2}}$$

**Figure 8:**
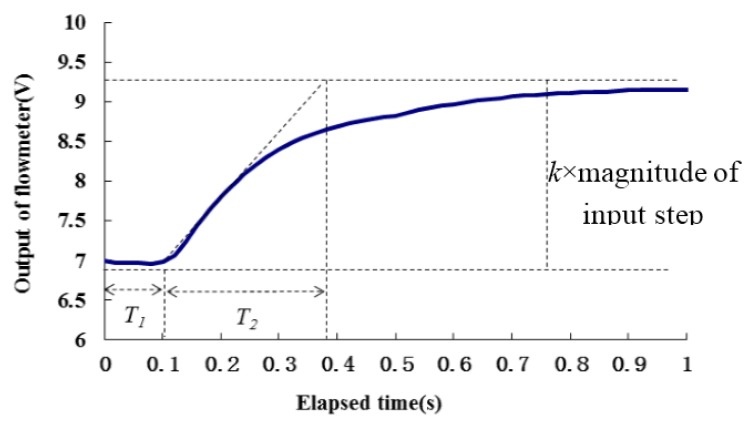
Open loop response to a step input *P* = 1200 μs, to obtain the transfer function coefficients.

The time constants *T_1_*, *T_2_* and the gain *k* were obtained experimentally from an open loop response of the system to a typical step input (input pulse width is 1200 μs, magnitude of flow rate change step is 19.21 mL/s,) as shown in Figure 8. The values are as follows: *T_1_* = 0.105 s, *T_2_* = 0.28 s, *k* = 0.117 mL^−1^·s·V. The Ziegler Nichols method suggested the best average settings for a PID controller with transfer function:

(5) $$G(s)=K_{c}(1+\frac{1}{T_{i}s}+T_{d}s)$$

where $K_{c}=\frac{1.2T_{2}}{{kT}_{1}}$, *T_i_* = 2*T*_1_, *T_d_* = 0.5*T*_1_.

The above values of *T_1_*, *T_2_*, *k* gave approximatively:

*K_c_* = 27, *T_i_* = 0.21 s, *T_d_* = 0.057 s

**Figure 9:**
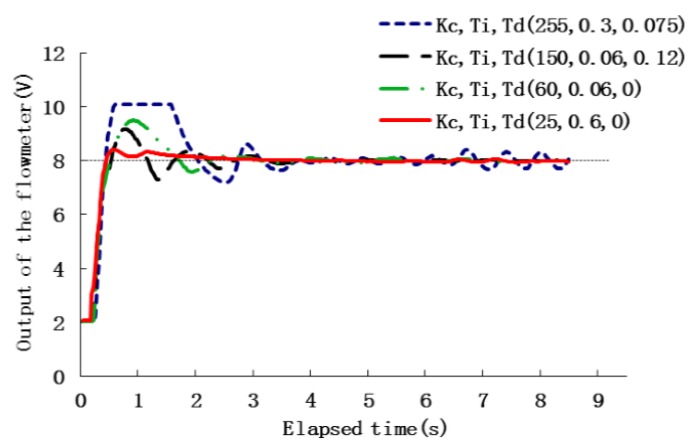
Variation in output of flowmeter as a function of time as flow rate controlled with four groups of different PID parameters, the set-point flowmeter = 8 V (equivalent to chemical flow rate = 11.8 mL/min).

Figure 9 shows the performance of the closed-loop control to a step input for four different groups of PID parameters when the set-point output of the flowmeter was 8 V (equivalent to a set-point flow rate = 11.8 mL/min). The Ziegler Nichols controller design method gave a reasonable first estimate of controller coefficients, through slight adjustment, PID parameters (*K_c_* = 25, *T_i_* = 0.6, *T_d_* = 0) was verified as most suitable for the system, they caused a smallest overshoot as well as least oscilation of the flow rate (the solid line) than others whoses PID parameters were tuned randomly, although each group of PID parameters could ultimately stablize the flow rate. Comprehensively comparing the index of these four transient responses, PID parameters (*K_c_* = 255, *T_i_* = 0.3, *T_d_* = 0.075) caused the transient flow rate to exceed the measurement range of the flowmeter, so it can not be used in the controller. The parameters (*K_c_* = 25, *T_i_* = 0.6, *T_d_* = 0) were preferred, as it only took roughly 1.3 s for the system to achieve a target flow rate with a very small overshoot.

### 3.5. Two-Phase Control

**Figure 10:**
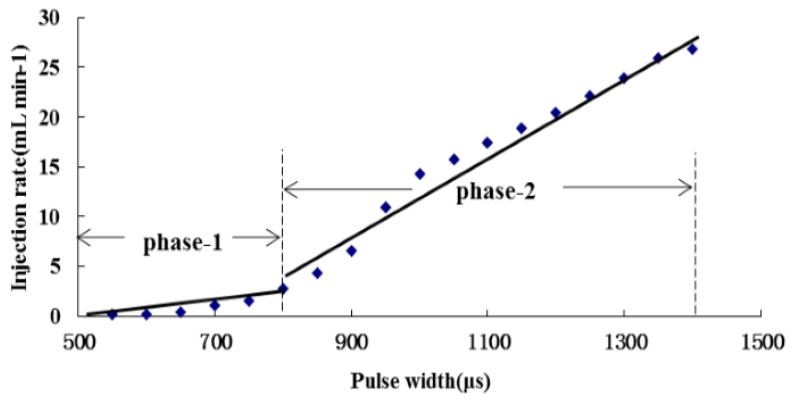
Sectional linear input-output characteristic of RRV.

The PID controller worked well for linear systems, however, due to the fact the input-output characteristics of the RRV are not strictly linear as shown in Figure 10, only one PID control module is not enough for precisely metering chemical flow rates in the entire range. The system could be treated as a partial linear system with two sections, where each section has different input-output characteristics. Section 1 is the scale when the pulse width is below 850 μs, under which conditions the injection rate is lower than around 4.5 mL/min, and Section 2 is when the pulse width is above 850 and the corresponding injection rate is higher than around 4.5 mL/min. In each section, the relationship between input pulse width and output injection rate was simplified as linear. By this simplification, the efficient control performance in entire range could be maintained through adding another PID module to the controller for Section 1.

**Figure 11:**
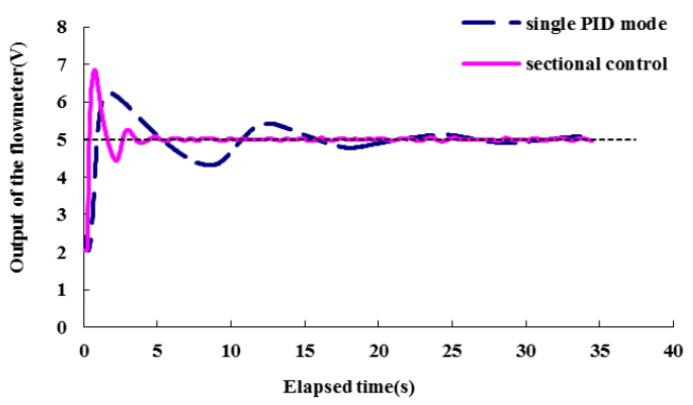
Time domain responses of the system, when the set-point flowmeter output is 5 V (equivalent to a chemical flow rate of 0.17 mL/min).

The tuned PID module with parameters (*K_c_* = 25, *T_i_* = 0.6, *T_d_* = 0) was adequate for Section 2, and it worked well when the set-point output of the flowmeter was above 7 V (equivalent to a set-point chemical flow rate higher than 4.5 mL/min), but it was not suitable for Section 1. As the target output of the flowmeter was below 7 V, the time needed to achieve the target flow rate was much longer. An example for the set-point output of the flowmeter at 5 V (equivalent to a set-point chemical flow rate = 0.17 mL/min) is shown in Figure 11 (dashed line), and the time delay is *ca.* 15 s.

Based on the analysis above, the control strategy was modified to a two-phase controllor with two different PID modules for the two sections, respectively. Section 2 continued to use the tuned PID module, and another PID module with parameters (*K_c_* = 70, *T_i_* = 0.2, *T_d_* = 0) tuned by the Ziegler Nichols method as well was used for Section 1. As a result, the flow rate control in the range of less than 4.5 mL/min was significantly improved. Figure 11 also shows an example of the set-point flowmeter output at 5 V (equivalent to a chemical flow rate of 0.17 mL/min) metered by the two-phase controller (solid line). It took less than 4 s to achieve a steady state, which is a great improvement compared with the dashed line obtained from a single PID mode.

Table 1 shows indexes of transient responses regulated by the two-phase PID controller. Overall, the steady-state errors are less than 0.021 V, meaning that flow rates can be metered and stabilized at the targeted value precisely. The settling times are less than 3.17 s, indicating the controller could regulate the flow rate in a short time. Associating the response index with the two sections, it can be found that the settling times for Section 2 (0.98~1.31 s) are less than those for Section 1 (2.58~3.32 s), and the maximum overshoot values (4.13%~6.48%) for Section 2 are less than those for Section 1 (20.77%~36.88%) as well. This illustrates that the PID module for Section 2 meters the flow rate more efficiently than the module for Section 1. It does not mean the module parameters (*K_c_* = 70, *T_i_* = 0.2, *T_d_* = 0) for Section 1 is not the best fitted, because many other PDI modules with various parameters had been tried, and the parameters (*K_c_* = 70, *T_i_* = 0.2, *T_d_* = 0) worked the best.

**Table 1:** Indexes of the transient responses.

Set-Point Voltage of Flowmeter (V)	Equivalent Flow Rate (mL/min)	Settling Time (s)	Steady-State Error (mL/min)	Maximum Overshoot (%)	Rise Time (s)	Phase NO.
4	0.129	3.09	0.011	24.65	0.42	1
5	0.174	3.17	0.016	36.88	0.40	1
6	0.277	2.58	0.037	20.77	0.42	1
6.5	2.611	3.32	0.073	30.98	0.44	1
7	4.647	1.31	0.059	6.48	0.38	2
8	11.809	0.98	0.054	4.13	0.38	2
9	23.367	1.26	0.063	5.36	0.36	2

### 3.6. EC Measuring Method to Evaluate the Effect of the Closed-Loop Control

**Figure 12:**
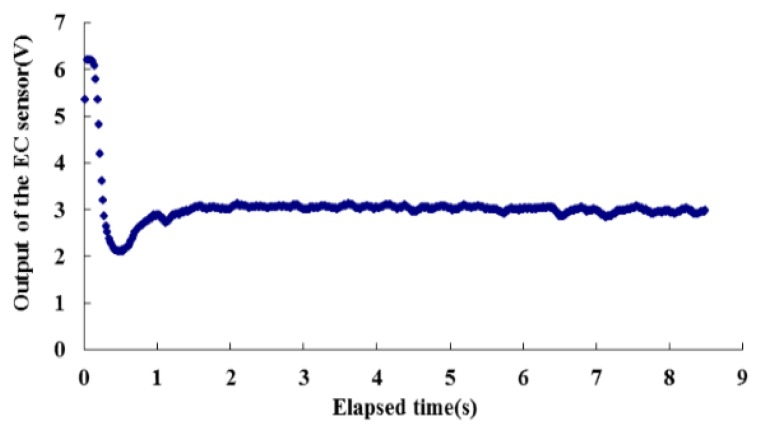
Output of the EC sensor when the desired output of the flowmeter was set at 8 V (equivalent to a desired chemical flow rate set at 11.8 mL/min).

The performance of the closed-loop controller also can be verified from the output of the EC sensor as shown in Figure 12, in which the readings are from the same test displayed by a solid line in Figure 9. Comparing these two curves, the flowmeter output and EC sensor change simultaneously. Although they have opposite trends (the calibration of the EC sensor is negative correlative, the higher the flow rate and therefore the conductivity, the lower the output voltage), both could be used to indicate the flow rate. After the flow rate achieved a steady state, the EC values were analyzed. The consistency and stability of the EC values indicate the concentration of the salt solution was stable, hence the flow rate metered by the closed-loop controller was constant.

## 4. Conclusions

An accurate control of chemical injection rates is the basis of the site-specific, variable-rate application of chemicals. This article tested a feasible method to control the flow rate for a direct nozzle injection system. From the results, the following conclusions can be drawn:

1. The RRV used in the DNIS could inject chemical with flow rates ranging from 0.1 mL/min to more than 30 mL/min, which covers most spraying operations in the field.
2. The thermodynamic flowmeter met the requirement of measuring wide range of flow rate, and it was independent of chemical viscosity. However, it was affected by the thermal conductivty of the liquid medium, so calibration for different chemicals was necessary before application.

The closed-loop controller (PID) worked well for metering and controlling flow rates precisely, but the tuning of PID parameters should be carefully considered, as it was highly related to the input-output charicteristics of the RRV. If the input-output characteristics of the injection unit are non-linear, a two-phase controlling method was an optimal solution.
